# The quiet rest of the warrior: a story of life, death, taphonomy and bone diagenesis

**DOI:** 10.1007/s12024-026-01177-x

**Published:** 2026-02-16

**Authors:** Edda E. Guareschi, Paola A. Magni, Brendan Chapman, Davide Persico

**Affiliations:** 1 South Tyrol Museum of Archaeology, 39100 Bolzano - Bozen, Italy; 2 Medical, Molecular and Forensic Sciences, Murdoch University, Murdoch, WA 6150 Australia; 3 The UWA Oceans Institute, The University of Western Australia, Perth, WA 6009 Australia; 4 Dipartimento Di Scienze Chimiche Della Vita E Della Sostenibilità Ambientale (SCVSA), Università Degli Studi Di Parma, Parma, Italy

**Keywords:** Fluvial taphonomy, Forensics, Museum collections, Postmortem interval (PMI), Postmortem history

## Abstract

The abundance of human skeletal remains housed in museum collections can promote multidisciplinary expert collaborations and training following forensic protocols and methodologies. These can assist in the reconstruction of part of the history of the ecofacts, add to the cultural heritage and highlight the scientific and social value of museum collections. This study aims to present a typical example. A human skull recovered in the late 1960s / early 1970s in the Po River alluvial plain, in northern Italy, has since been housed in a local ethnographic museum, the Fondazione Museo Ettore Guatelli (https://www.museoguatelli.it/en/). Recently rediscovered, the skull shows several morpho-anatomical alterations suggestive of congenital disease and perimortem trauma, along with peculiar bone staining of taphonomic and diagenetic origin. Despite remarkable limitations, including the skull fragility, an international multidisciplinary research team was able to apply modern protocols of forensic investigations to determine the biological profile, as well as a pathological condition, the cause of death (non-natural), the postmortem interval (PMI) (late Middle Ages) and the postmortem taphonomic history, reincluding the individual into the cultural narrative of the local community.

## Introduction

**Fig. 1:**
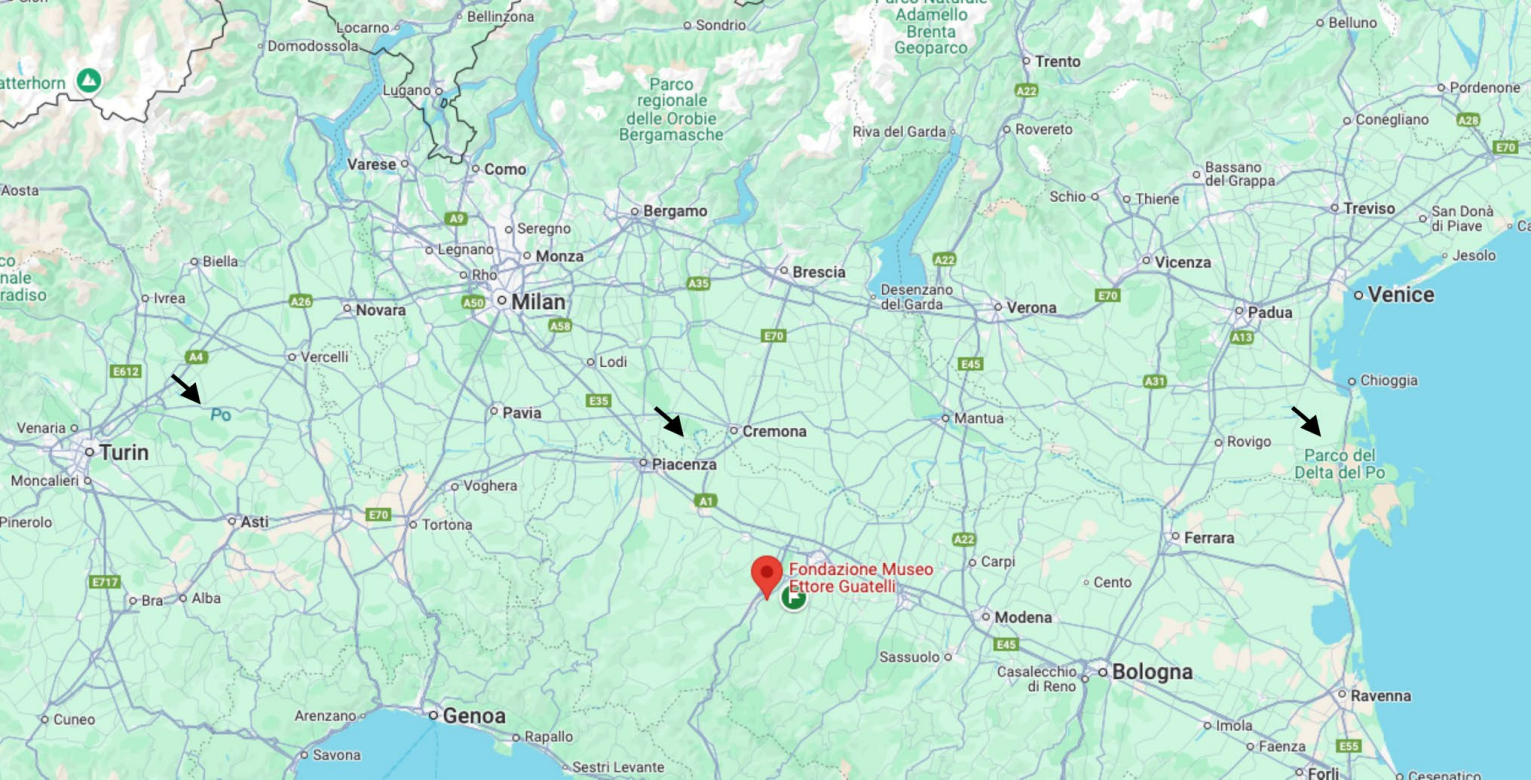
Po River area in the north of Italy (44°58′7.19" N 12°32′29.39" E) with the locations of the river source and the delta, the estimated site of the skull recovery (solid arrows) and the Fondazione Museo Ettore Guatelli (pinpoint icon). The Po River spans 652 km (405 mi) and has an average discharge at its outlet of approximately 1200 m^3^/s

Museum collections often house human skeletal remains, that serve as invaluable records of human biology, history and cultural heritage. Their collection is generally documented, however, they are not always comprehensively analysed with the aim of reconstructing the biological profile and the lifestyle of the living individuals, as well as the circumstances of their death and their postmortem history. This can be achieved by identifying diet and migration patterns, as well as evidence of pathology, trauma and taphonomy [1]. Beside the cultural, scientific and social relevance of a comprehensive skeletal analysis, the overall process can constitute an efficient and focused training for forensic practitioners investigating human skeletal remains, since the overlap of materials, methods, analytical techniques and data interpretation is substantial [2–4]. An example is observed in the case of a human skull recovered in the late 1960 s or early 1970 s in the Po River area near Parma, in northern Italy (Fig. 1). The skull was donated to a local ethnographic museum, the present Fondazione Museo Ettore Guatelli [5], where it was included in its extensive collection of artifacts and ecofacts. Following a recent archival review, a multidisciplinary international and interinstitutional team gained the opportunity to analyse the skull using several advanced techniques also used in forensic investigations. The aims of the analysis were to reconstruct the individual’s biological profile, investigate their life history and the circumstances of death, and examine the postmortem alterations processes occurred between death and the recovery.

## Materials and methods

**Fig. 2:**
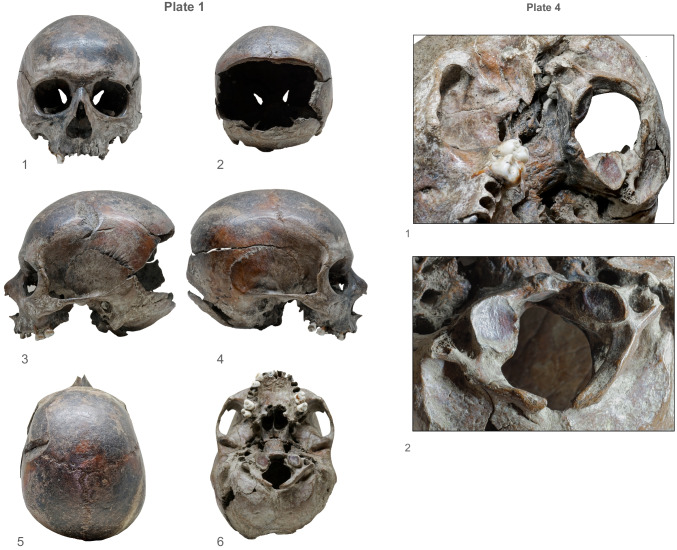
**A** Macroscopical appearance of the skull in the coronal, sagittal and axial planes. **B** atlanto-occipital assimilation, a rare congenital anomaly of the craniovertebral joint in which the first cervical vertebra is fused to the condylar part of the occipital bone

A human skull with complete splanchnocranium, except for the missing mandible, and incomplete neurocranium (Fig. 2A) was recovered by a community member in the Po River area near Parma (Italy) between the late 1960 s and early 1970s. No further information is available to date, including any assessment or investigation by law enforcement, any other associated human remains, or any cleaning, conservation or preparation procedures, e.g., to remove debris or colonizing fauna. The inclusion in the collection of the Fondazione Museo Ettore Guatelli shortly followed the recovery, and a recent archival review identified the need of more information as a priority.

The fragility of the skull discouraged any transportation. High-resolution images were captured by digital photography (Canon PowerShot SX70 HS) prior to any invasive procedures, e.g., sampling.

The individual’s biological profile, pathology, and trauma were assessed on-site according to established protocols [3, 6–8], two teeth were then carefully extracted using sterilised dental extraction forceps and stored in plastic screw cap laboratory containers. Additionally, tiny bone fragments were collected with clean pliers from the left transverse process of the first cervical vertebra (C1, or atlas). The total amount of biological material removed from the skull did not exceed 20 g.

The left upper first premolar (24) was examined at Murdoch University (Western Australia) for DNA isolation and assessment of quantity and quality of the DNA extract. Prior to DNA isolation, the tooth was wiped using a 10% bleach solution to remove any exogenous biological material associated with handling prior to the laboratory. The outer surface of the tooth was then removed using a rotating tool (Dremel®) with sterilised diamond wheel bit (P/N 7104 and 7123). The powder generated was discarded, assumed contaminated. The tool was operated using a slow speed setting to ensure that minimal heat was generated. Following cleaning, a cavity was ground from the bite surface of the tooth to expose the tooth pulp which was powdered and collected. Two powder samples were generated from the tooth, a sample containing mostly pulp and surrounding bone and a sample of the powdered bone remaining after removal of the pulp. Both powder samples were processed for DNA extraction using a modified DNA Investigator tooth protocol with overnight incubation at 56’C and elution in 50uL [9]. Assessment of the isolate was performed using the Applied Biosystems Quantifiler Trio on a QuantStudio 6 real time PCR instrument. This enabled parallel assessment of DNA quantity, degradation and sex determination.

The left upper second premolar (25) was radiocarbon dated (^14^C) at the Centro di Datazione e Diagnostica (CEDAD) at the University of Salento (Italy) by Accelerator Mass Spectrometry (AMS). The bone fragments were transferred to the conservation laboratory of the Shipwrecks Museum (Western Australia) for histological analysis by light microscopy (Leica MZ6 StereoZoom optical microscope. Image processing software Leica Application Suite Version 4.12.0) on thin undecalcified sections, and analysis of taphonomy and bone diagenesis by Attenuated Total Reflectance (ATR) in Fourier Transform Infrared (FTIR) spectroscopy and X-Ray Fluorescence (XRF).

Finally, approximately 3 g of soil embedded in a dental alveolus and in the left ear canal were sampled using a dental excavator and stored in a clean vial. The soil samples were sent to the laboratory of the Dipartimento di Scienze Chimiche della Vita e della Sostenibilità Ambientale (SCVSA) at the University of Parma (Italy) for sedimentary analysis.

The interpretation of the taphonomic alterations of the skull considered the depositional environment in which the remains were found, as it affects the biomineralized tissues of the bone and teeth [10–12]. Specifically, the climate of the Po River valley in the north of Italy (44°58′7.19" N 12°32′29.39" E) is predominantly humid subtropical/hot summer temperate (Köppen-Geiger climate classification: Cfa), with annual precipitation around 800 mm. The Po River, which spans 652 km (405 mi), has an average discharge at its outlet of approximately 1200 m^3^/s [13, 14].

**Fig. 3:**
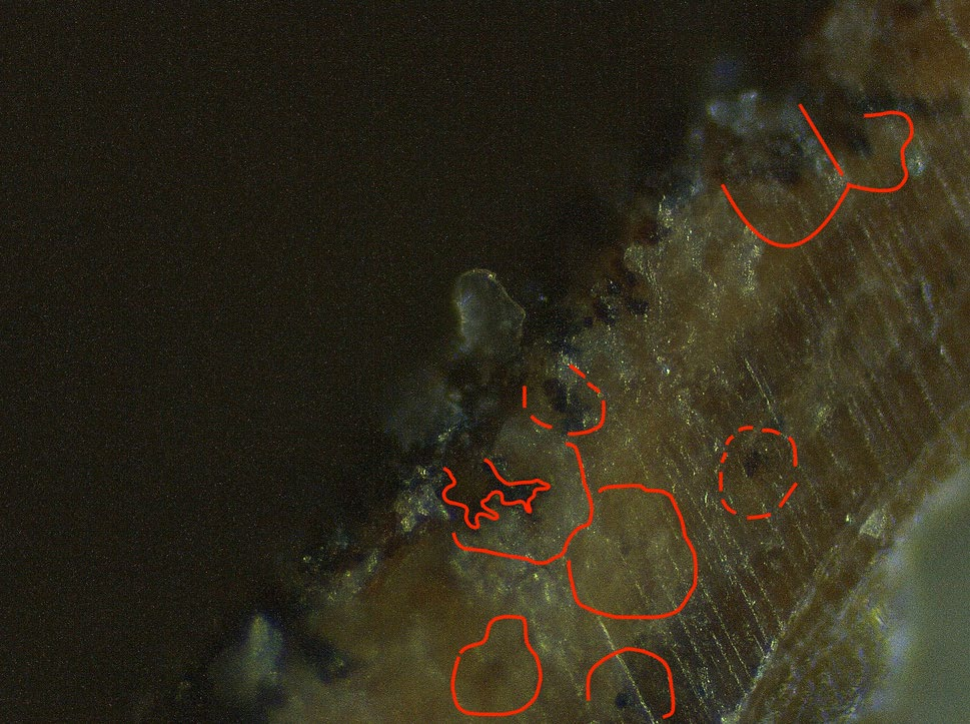
Histological detail of an undecalcified thin section observed by light microscopy (200X). The preservation of the bone microstructure is very poor (OHI 0–1), with only Haversian canals and some outlines of cement lines (drawn borders) identifiable

**Fig. 4:**
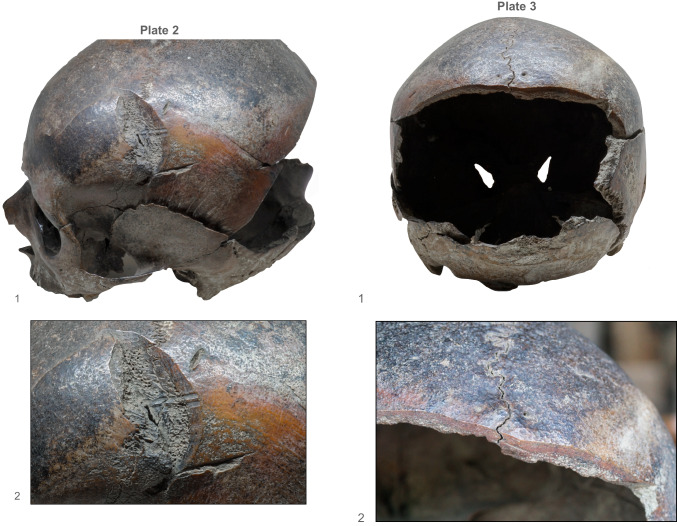
**A** **B** Multiple cranial fractures in the left fronto-parietal region and in the bilateral parieto-occipital region. Their morphology is consistent with the action of a heavy tool with a sharp edge (e.g., axe or sword)

**Fig. 5:**
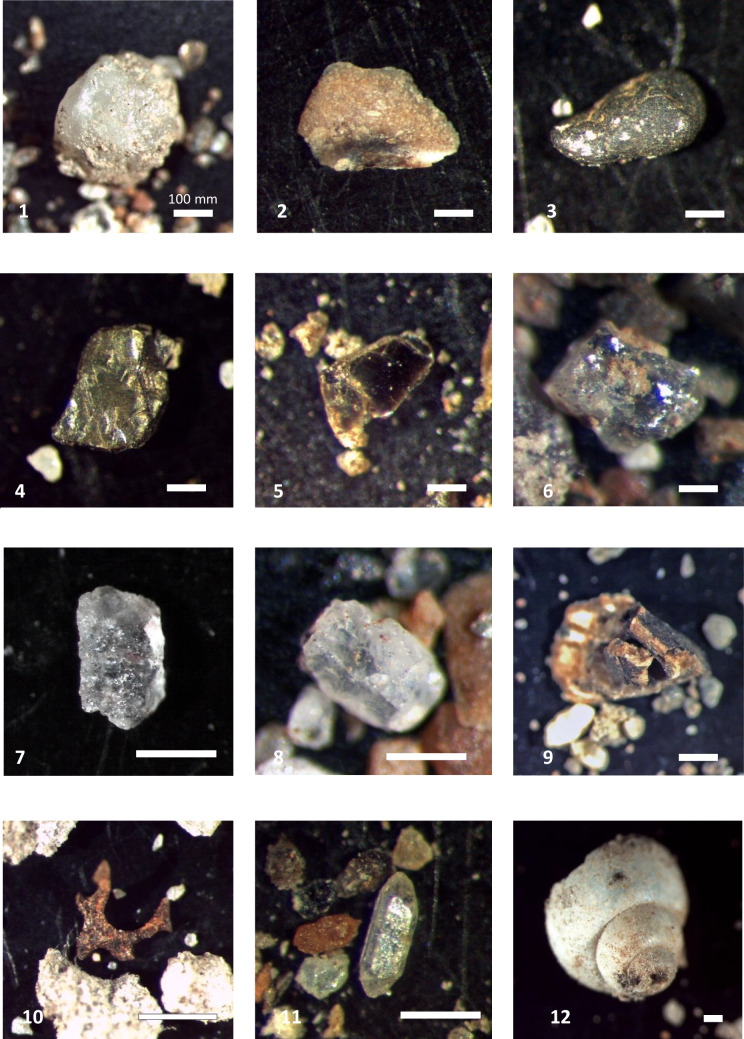
Sedimentary analysis of the soil collected from the skull’s natural cavities: 1: Quarzite; 2: Manganocalcite; 3: Pirite; 4: Muscovite; 5: Biotite; 6–8: Clear quartz; 9: Apatite (bone) with sulfides and iron oxides; 10: Hematite, limonite (internal model of spongy bone cavity); 11: Clear quartz crystal; 12: Freshwater mollusc shell [Gastropoda (CUVIER, 1797)] (100X)

The type of analyses performed, the applied analytical techniques, and the related results are detailed in Table 1. Figures 1, 2, 3, 4 and 5, show a selection of relevant visual details.

**Table 1:** Summary of the details and the related results of the macroscopical, microscopical and chemical analyses performed on the skull and/or on the collected samples

Type of Analysis	Sample	Details and applied analytical techniques	Results
Biological Profile	Skull	Species: macroscopic morphological anatomy	*Homo sapiens*
	Skull, left upper first premolar (24)	Sex: morphological anatomy, confirmed by genetics (Y-chromosome)	Male
	Skull, bone fragments of the left transverse process of the first cervical vertebra	Age estimation: odontology, confirmed by histology	Young adult
	Skull	Population affinity: morphological anatomy	Caucasoid
Pathology	Skull	Anatomical Pathology	Atlanto-occipital assimilation
Trauma	Skull	Fracture morphology	Multiple cranial fractures by hacking trauma (“chop injuries”)
Postmortem Interval (PMI)	Left upper second premolar (25)	^14^C dating	68.2% probability 1409–1443 CE
	Left upper second premolar (25)		95.4% probability: 1326–1352 (5%) 1393–1460 CE (89.9%)
Taphonomy and Diagenesis	Bone fragments of the left transverse process of the first cervical vertebra	Histology	OHI 0–1
	Skull	Bone preservation and diagenesis (FTIR)	Rare to no fluvial flotation and transport
	Bone fragments of the left transverse process of the first cervical vertebra		CI 3.58 (early fossilization) calcite/carbonate ratio 0.43–0.47
	Bone fragments of the left transverse process of the first cervical vertebra	Trace Elements (XRF)	Bulk concentration peaks of Fe, Mn, Zn

## Results

### Biological profile

The skull macroscopical anatomy identifies the modern *Homo sapiens* species, with the evolutionary timeline confirmed by radiocarbon dating (late Middle Ages). Sex and age were also estimated by macroscopical anatomy, including that of the maxillary dental arch and dentition [15], and confirmed by genetics (successful Y-chromosome target amplification for the DNA isolate) and histology. Genetics showed heavily fragmented male DNA in very low amount. The presence of several primary osteons, observed by histology, supports the early stage of adulthood [16, 17]. An evident nasal sill suggests the affinity to the caucasoid population [15].

### Pathology and traumatology

The individual carried a rare congenital anomaly of the craniovertebral joint, known as atlanto-occipital assimilation, in which the first cervical vertebra is fused to the condylar part of the occipital bone (Fig. 2B). According to multiple studies, the incidence of atlanto-occipital assimilation ranges between 0.14% to 0.75% of the population, and the prevalence ranges between 0.08% to 2.76% [18–20]. Multiple cranial fractures were observed as having occurred in the perimortem period. The morphology of the extensive multiple fractures in the left fronto-parietal region and in the bilateral parieto-occipital region is consistent with the action of a heavy tool with a sharp edge, such as an axe or a sword inflicting chop wounds, also known as hacking trauma [21–23] (Fig. 4).

### Postmortem Interval (PMI)

Dating was achieved by radiocarbon (^14^C) analysis, the individual’s death was estimated to have occurred as either in 1409–1443 CE (68.2% probability) or in 1326–1460 (95.4% probability, divided in 1326–1352, 5% and 1393–1460 CE, 89.9%).

### Taphonomy and diagenesis of the remains

Nine well-preserved teeth still articulated to their alveolar processes of the maxillary bone indicate rare to no fluvial flotation and transport [24–26]. Other taphonomy findings include a prevalent grey-brown colour of bone, with interspersed areas of orange hues, and the presence of abrasion, inclusions, infiltrations and microfissures. The thin undecalcified histological sections showed very poor preservation of the bone microstructure, with only Haversian canals and some outlines of cement lines identifiable, the corresponding value of the Oxford Histological Index (OHI) is 0–1 [27] (Fig. 3). The increase in bone crystallinity and the calcite/carbonate ratio (CaCO₃/CO₃^2^⁻), quantified by ATR-FTIR, the presence of specific trace elements, detected by XRF, and the presence of hyaline quartz, manganocalcite and arenaceous clasts, iron sulphide nodules and iron oxides embedded in the skull’s natural cavities confirm a long burial in the alluvial sandy sediment of the Po River. The submersion is further confirmed by fragments of freshwater mollusc shells [Gastropoda (CUVIER, 1797)] in the embedded material (Fig. 5).

A summary of the results is presented in Table 1.

## Discussion

This case was investigated following a thorough forensic protocol, constituting an example of ideal training for forensic practitioners. The young male adult died in the first half of 1400 CE because of interpersonal violence. Considering the multiple limitations of this case study, it is reasonable to suppose that the body was deposited in the floodplain area of the Po River, where it decomposed until skeletonization, disarticulation and scattering of the isolated remains within the fluvial sediment. The individual’s skull was recovered approximately six hundred years later, and no historical information is available today, except that it joined the museum collections through an act of donation. Given the extended PMI, achieving a positive personal identification was highly unlikely. However, it was possible to identify the individual as a caucasoid suffering of atlanto-occipital assimilation (sometimes also referred to as atlanto-occipital fusion or atlas occipitalization, atlas assimilation, and occipitocervical synostosis), a rare congenital pathological condition involving the partial or complete fusion of the first cervical vertebra with the occipital bone [18–20]. The etiology of atlanto-occipital assimilation is complex and can arise from congenital and environmental causes, including cultural practices of artificial cranial modification in archaeological populations [28]. Atlanto-occipital assimilation can be asymptomatic, especially in the young, or can include neurological symptoms, such as neck pain with movement limitation, muscular weakness and lack of movement coordination (ataxia) which worsen with age and can lead to sudden death due to the compression of the brainstem. Atlanto-occipital assimilation can also be associated with Klippel-Feil syndrome, another congenital condition in which 2 or more cervical vertebrae are fused. The physical appearance of both atlanto-occipital assimilation and Klippel-Feil syndrome is evident, with very short neck and low posterior hairline.

The death of the young individual was clearly non-natural. The multiple anatomical locations, the number, the severity and the macroscopic morphology of the cranial lesions indicate a voluntary homicidal act, possibly achieved by multiple assailants, by a combination of blunt and sharp force repeatedly applied to the left fronto-parietal and the bilateral parieto-occipital anatomical regions. The wide V-shaped lesion marks with straight defined kerf floor, radiating fractures and bone loss suggest the use of one or more heavy tools with a sharp edge, possibly identifiable in cleavers, axes or massive swords. Such highly energetic actions produced multiple and deep cranial fractures (“chop injuries”) rapidly leading to acute post-traumatic haemorrhagic shock. The taphonomic and diagenetic analysis of the skull revealed a geochemical profile consistent with prolonged burial in the alluvial sandy sediment of the Po River. The grey-brown staining with intense orange patches is consistent with environmental exposure to fluvial sedimentary conditions, as described in previous studies [e.g., 29]. A small sample from the first cervical vertebra underwent pretreatment and analytical procedures according to well-known and accepted radiocarbon dating methods [30, 31], and after calibration to calendar years, the highest probability of the individual’s life span was determined in the first half of 1400 CE (late Middle Ages).

The macroscopical and microscopical preservation of bone were not consistent. Interestingly, the bone destruction usually associated to fluvial flotation or transport [24, 32] was absent, with excellent preservation of the splanchnocranium (or facial skeleton), the maxillary dental arch and several teeth (Fig. 2A). On the contrary, the histological analysis revealed a very poorly preserved microstructure, only characterized by Haversian canals and sparse outlines of cement lines identifiable (OHI 0–1). Combined with the widespread presence of mineral inclusions [33], these findings suggest an early stage of bone fossilization through permineralization. This conclusion is further supported by the Crystallinity Index (CI) of bioapatite, measured at 3.58 (reference for fresh bone: 2.8–3.0) [34], indicating increased structural order due to crystal growth, and by a relatively high calcite/carbonate ratio (0.43–0.47, compared to a control fresh bone value of 0.34), indicating the presence of calcite and the leaching of carbonate ions [35, 36]. Additionally, peaks in the bulk concentrations of Fe, Mn, and Zn observed in the XRF spectrum corroborated this process.

The physical and mineralogical characteristics (*“facies”*) of the skull are consistent with the typical alluvial fossils of the Po Valley [37]. Such fossils, partially or completely mineralized, constitute a thanatocoenosis characterized by faunal remains belonging to different geological epochs, from the Pleistocene to the Holocene. Their most represented component are mammalian bones [38–40], usually isolated after disarticulation and exposed to fluvial transport due to environmental erosion. Cranial bones may retain some degree of articulation, especially in adult individuals in which the cranial sutures are partially or entirely fused, or where oxidized sulphides act as a cementing agent. The most common fossilization pattern in the Po River alluvial plain is permineralization with pyrite, manganocalcite and manganese minerals [29, 41]. The natural bone cavities of alluvial fossils, including trabecular bone when exposed, show the accumulation of sediment with clasts of diverse dimensions, from submillimetric to a few centimetres. In the present case study, a freshwater gastropod shell was found enclosed in such accumulated material, further supporting the fossil’s origin within an alluvial environment.

## Conclusions

After a six-hundred-years (est.) deposition in the alluvial sediment of the Po River, as well as over fifty years in a museum collection, the life, death and postmortem history of a young man was reconstructed by a multidisciplinary international research team through the analysis of his skull and teeth in the early stage of fossilization. He carried an evident physical disability, lived around the first half of 1400 CE and died because of interpersonal violence. In the absence of any historically reliable documentation, it is not possible to reconstruct the circumstances of the social conflict. Despite significant limitations, e.g., the requirement of minimally destructive sampling, this case study highlights critical issues about the study of human remains housed in museum collections, including but not limited to engagement in ethical research [42–44] and recognition of their scientific and social relevance, also achieved by allowing training for forensic practitioners [45–47].

## Data Availability

The supplementary material is available from the corresponding author upon reasonable request.
